# The Efficacy of FlexMaster’s IntroFile, PreRaCe and Gates Glidden Drills in Straight-Line Access: A CBCT Assessment

**Published:** 2014-07-05

**Authors:** Narges Farhad Mollashahi, Mahdi Sohrabi, Leila Farhad Mollashahi, Mojdeh Mehdizadeh

**Affiliations:** a Department of Endodontics, Dental School, Zahedan University of Medical Sciences, Zahedan, Iran; b Department of Oral and Maxillofacial Radiology, Dental School, Zahedan University of Medical Sciences, Zahedan, Iran; c Department of Oral Medicine, Dental School, Zahedan University of Medical Sciences, Zahedan, Iran; d Department of Oral and Maxillofacial Radiology, Dental School, Isfahan University of Medical Sciences, Isfahan, Iran

**Keywords:** Access Cavity, CBCT, Cone-Beam Computed Tomography, Engine-Driven Instruments, Radicular Access, Root Canal Preparation, Rotary Systems, Straight-Line Access

## Abstract

**Introduction:** An overlooked but important part of successful root canal treatment is a straight-line access (SLA). The purpose of this *in vitro* study was to compare the efficacy of IntroFile and PreRaCe rotary instruments with Gates Glidden (GG) drills in gaining SLA by cone-beam computed tomography (CBCT). **Methods and Materials:** A total of forty five extracted mandibular first molars were selected and mounted in dental like arches. Subsequently, they were randomly classified into three groups (*n*=15). After preparation of a standard access cavity, orifices of the mesiobuccal canal was reached and a #10 file was inserted to explore the canals until the file tip was visible at the apex. Then, preoperative CBCT images were taken. SLA was gained in three groups; group 1, FlexMaster’s IntroFile (FM); group 2, PreRaCe (RC) and group 3, GG. Again, the first binding file at the working length (WL) was placed in the canal and postoperative CBCT images in similar positions were taken. The pre/post operative morphology of the canal was evaluated for changes. Data was analyzed using the one-way ANOVA and post-hoc Bonferroni analysis. **Results:** The average amount of reduction in coronal canal curvature in FM, RC and GG groups was 2.43±1.79, 3.17±2.05 and 8.7±3.45, respectively. This descending trend was statistically significant. The difference between pre/post SLA changes in FM and RC groups was significant compared to GG group, while there were no significant differences between RC and FM. **Conclusion:** GG drills produced extraordinary results in reducing coronal curvature of the canal and achieving SLA. They are also more effective than nickel-titanium (NiTi) rotary instruments in canals with coronal curvature.

## Introduction

Undoubtedly, preparation of a straight-line access (SLA) from the canal orifice to the apical extend of the canal is imperative for root canal preparation using cutting instruments. Under ideal circumstances, these instruments should reach the apical foramen or at least the first curvature of the canal without bending. Excessive bending of the instruments, which results in inappropriate control, can lead to numerous problems. Without having SLA, attempts for cleaning and shaping of the canal will usually result in procedural faults such as ledging, transportation and zipping. In contrast, an instrument which does not bend during operation enables a better sense of canal anatomy and improves the quality of file performance in root canal system [1]. 

The term SLA describes a preparation pattern that provides a straight or outwardly flared, unimpeded path from the occlusal to the apical end or the first curvature of the canal. This allows the file to reach the apex with minimal deflection. The logical consequence is that the root is treated without risk. SLA involves the selective removal of the tooth structure from outer canal walls to protect the furcal surface. Different instruments have been suggested for gaining SLA, including various burs, Gates Glidden (GG) drills and nickel-titanium (NiTi) rotary instruments [1-4].

**Figure 1:**
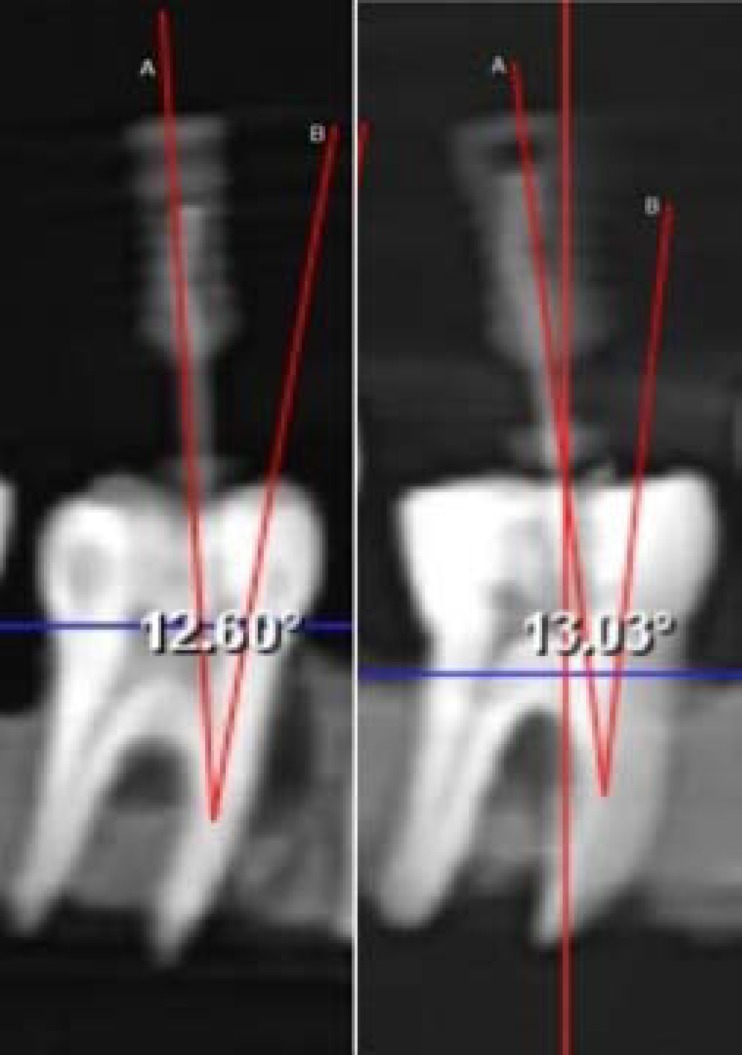
Pre-procedural images for calculation of the canal coronal curvature (angle between the lines); *A)* file long axis and *B)* the line drawn from NiTi marker to the beginning of canal coronal curvature

By using microcomputed tomography, Peters *et al*. [5] have shown that significant portions of canals are left untouched during instrumentation due to the cross sectional irregularities and curvature of the canals. Their study emphasized on the necessity of SLA. Mannan *et al.* [6] stated that SLA provides the best chance of debridement and cleaning of the entire canal space. Many studies have investigated the relation between coronal flaring and removal of the interferences from the coronal third of the canal and changes in the working length (WL), actual diameter of apical region as well as the diameter of the first binding file (FBF).

Schroeder *et al.*, Sadeghi *et al. *and Dean Davis *et al.* have independently investigated the changes of WL before and after making corono-radicular access using NiTi and stainless steel (SS) files [4, 7, 8]. The results indicated the reduction of WL in post-SLA condition. The reduction rate in the group which was prepared by SS files and GG drills was significantly higher than the group which was only prepared by NiTi instruments. The three mentioned studies emphasized on the importance of SLA and its effects on WL and avoidance of procedural accidents [4, 7, 8]. Tan and Messer stated that the type of used instrument for coronal and middle flaring had an impact on the first file size that binds at WL, *aka* the FBF [9]. Tennert *et al.* [10] showed a significant difference in the diameter of the first file fitting at the apex (FBF) before and after coronal preflaring with different instruments.

Currently, a new generation of x-ray imaging known as the cone-beam computed tomography (CBCT) is introduced [11-13]. Regarding the influence of instrument properties on canal transportation as well as the role of SLA on long-term prognosis of endodontic treatments on one hand, and the advantages of CBCT in examining samples (including high speed scanning, lower dosages of radiation, quick easy access to information, high resolutions of 3D images and no sample destruction) on the other, the present study has compared the capability of different instruments in obtaining appropriate SLA, by means of CBCT.

**Figure 2:**
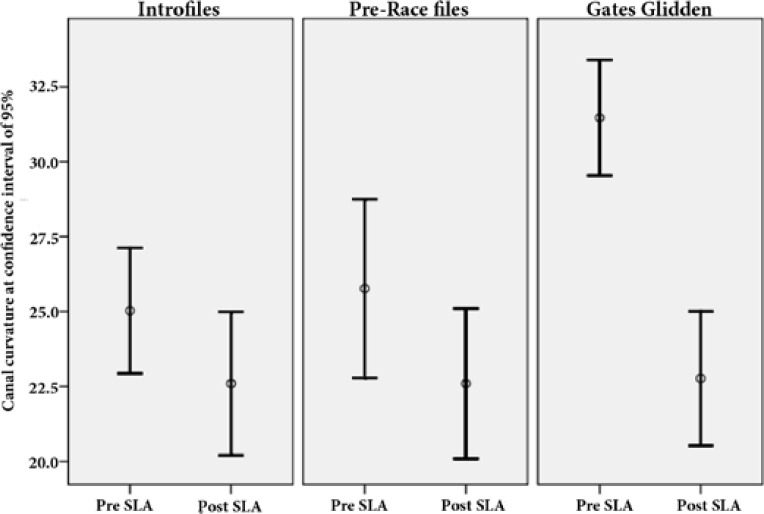
Mean value of canal curvature before and after straight-line access (SLA) preparation and the changes of canal curvature in three study groups [confidence interval (CI) = 95%]

## Methods and Materials

In this *in vitro* study, forty five extracted mandibular first molars with fully formed apices and the curvature of the mesial roots within the range of 10-20 degrees (according to Schneider’s method) [14] and two separate apical foramina, were collected and disinfected in 5.25% sodium hypochlorite (NaOCl). The samples were then kept in normal saline until the beginning of the examination. 

Every 7 to 8 teeth were mounted in an arc similar to dental arch in a way that their apices were visible. To determine the angle of curvature, a 1×1 mm index made of square NiTi orthodontics wire, was cut and attached to the tip of mesiobuccal cusp of each tooth. Standard access cavities were prepared. After access to the orifice of the mesiobuccal canal, a #10 K-file (Dentsply, Maillefer, Ballaigues, Switzerland) was introduced into the canal and directed towards the apex until its tip was seen at the apical foramen. To unify canal diameters, the teeth which allowed easy passing of file #10 were excluded from the study. Before starting to prepare the SLA, cross sectional sagittal CBCT images were taken with a file #10 inserted to the WL. Then, the images showing the file angles as well as the marker of mesiobuccal cusp tip were saved for comparison with post-SLA images (Figure 1).

Then, the access cavities were corrected using a high speed handpiece and an ISO No. 500 314 171 007 010 tapered fissure bur (Kerr Corporation, Orange, CA, USA) to remove the dentin shelves in the chamber and the coronal tooth structure. Teeth were randomly classified into three groups (n=15). In the first and second groups, coronal and middle flaring of the canal was performed using FlexMaster’s IntroFile (FM) (VDW, Munich, Germany) and PreRaCe file (RC) (FKG Dentaire, La-Chaux-de Fonds, Switzerland) installed on an electronic torque control unite (Endo IT professional, Aseptico Inc., Woodinville, WA, USA) set at speed/torque of 250 rpm/3 Nm and 500 rpm/1.5 Nm, for FM and RC files, respectively.

**Table 1:** Comparison of the changes in canal curvature in all study groups [COC=changes of canal curvature (degrees)]

	**N**	**COC Mean (SD)**	***P-*** **valu** ***e***
**FlexMaster**	15	2.42 (1.80)	0.001
**Gates Glidden**	15	8.7 (3.45)	
**PreRaCe**	15	3.17 (2.05)	

Flaring was accomplished based on the instructions provided by the manufacturer for each system. In the FM group flaring was performed using the IntroFile 20/0.11 in less than 3 mm of the WL and then the canal was flared using FM files 25/0.06 and 25/0.04. The canal flaring in RC group was done using instruments 40/0.10 and 35/0.08 up to the middle zone of the canal. In all groups each file was used only for flaring of five canals [10].

In the GG group the flaring of coronal and middle thirds of the canal was conducted as follows: Mesiobuccal canal was filled with 2.5% NaOCl solution. Then, GG drills (Dentsply, Maillefer, Ballaigues, Switzerland) sizes 4, 3 and 2 (ISO sizes 110, 90 and 70) were inserted into the canal in a crown-down fashion with speed of 10,000 rpm until a slight resistance was felt. Afterwards, by applying a small pressure on the instrument at a point away from the inner curve of the canal, the drill was pulled out [15].

In all groups post-SLA CBCT images were taken with a file that was bound in the canal and its tip was visible at the apex. The pre/post-SLA changes of coronal curvature of the canals were compared for the changes in the coronal file angle compared with the marker of mesiobuccal cusp. Data was analyzed using the one-way ANOVA and post-hoc Bonferroni analysis.

## Results

In order to select an appropriate test for data analysis, the distribution type of canal curvature was investigated by the Kolmogorov-Smirnov test. The results revealed that the distribution of canal curvature in the studied groups followed a normal pattern. Therefore, for comparing the changes of canal curvature in the three studied groups, one-way ANOVA and post-hoc pairwise comparison tests were employed, based on Bonferroni method.

Studying the changes in canal curvature (pre-SLA and post-SLA differences) showed that there was no significant difference between the three groups in terms of the changes in canal curvature. The pairwise comparison of the two groups indicated that there was a significant difference between GG (8.7 mm), FM (2.4 mm) and RC (3.17 mm) in terms of the changes in canal curvature (*P*<0.001) (Table 1). In other words, the rate of changes in canal curvature in the GG group was significantly higher than the other two groups. However, no significant difference was observed between FM and RC file; meaning that the rate of changes in canal curvature in these two groups was similar (Table 2, Figure 2).

## Discussion

The findings of this study indicated that the difference between GG with FM and RC was statistically significant. The average reduction of curvature was 8.7, 2.43 and 3.17 in GG, FM and RC groups, respectively. Since NiTi rotary files remain at the center of canal [16], the canal would be less straightened, which is in line with the data released by Bryant *et al*., and Thompson and Dummer [17-19]. By using NiTi rotary files to uniformly expand the coronal portion of a canal outward from its original anatomical position, the canal shape tends to move toward furcal danger zone. In contrast, due to its special physical properties, GG selectively brushes and cuts dentin on the outstroke [20]. Thus, GG is the choice to preflare the canal orifice(s), intentionally relocate the coronal aspect of a canal away from external root concavities, and toward the greatest bulk of dentin [20, 21]. Since the employed instruments have different specifications, canal shaping after preflaring was different in three groups [10].

Different studies have investigated the SLA and coronal preflaring and have confirmed their advantages. In separate investigations, Schroeder *et al.* and Dean Davis *et al.* stated that the more accurate the coronal preflaring, the less variations of WL after canal preparation [4, 8, 22]. However, Ibarrola *et al.* [23] stated that coronal flaring prior to determining the WL significantly improves the accuracy of apex locator function. Luiten *et al.*’s [24] study emphasized that transportation rate was at a minimum level in the preflared canals. Follahdoost *et al.* [25] stated that, by improving canal cleaning and tactile sense, canal preflaring reduces the possibility of zipping and, subsequently, overextension. This, in turn, reduces the post treatment pain and discomfort as well as probable failures.

The studies which have been carried out with the purpose of determining more accurate parameters for biomechanical preparation of apical one third, have confirmed the relationship between the cervical preflaring of root canal and more accurate estimation of initial apical file (IAF) [26]. Determination of IAF without cervical preflaring will result in size underestimation. Thus, regarding the results of this study, it is expected that canals preflared by GG drills show a minimum discrepancy between the diameter of IAF and the actual diameter of the canal [26]. This can probably be associated with GG properties, including alloy type, configuration and functioning method.

This study was done on the mesiobuccal canals of mandibular molars with small diameters and sophisticated anatomy. Nair *et al.* [27] showed that the mesiobuccal canal of mandibular molars is not easily accessible due to its unique anatomy. Different studies investigated the effects of different instruments on determining the accuracy of the IAF. Tennert *et al.* [10] compared RaCe rotary system, with ProTaper and FM and obtained the most accurate estimation by using the first system. Previous studies introduced the LA axxess burs (SybronEndo, Glendora, CA, USA) as the best instruments for achieving this goal [28].

**Table 2:** Bonferroni test for pairwise comparison of the changes in canal curvature in three study groups [Confidence Interval (CI)=95%] (I=Marker of the long axis before SLA, J=Marker of the long axis after SLA; FM= FlexMaster, GG= Gates Glidden, RC= PreRaCe)

**I**	**J**	**I-J (Mean)**	***P*** ***-*** **value**	**Lower Bound**	**Upper Bound**
**FM**	GG	-6.273*	0.00	-8.58	-3.96
	RC	-0.746	1.00	-3.06	1.57
**GG**	FM	6.273*	0.00	3.96	8.59
	RC	5.526*	0.00	3.21	7.84
**RC**	FM	0.746	1.00	-1.57	3.06
	GG	-5.526*	0.00	-7.84	-3.21

As a diagnostic method, CBCT is considered a revolution in investigation and solving endodontic problems. By providing the possibility of viewing a region in three spatial plans as well as observing anatomic relationship between components, clinicians could have more predictable treatment strategies, although it is costly [11-13, 29]. CT scans decrease the potential of radiographic or photographic transfer error [30]. This study investigated the variations of canal coronal curvature using CBCT. Computer-aided grading of coronal curvature could reduce probable faults and could make it possible to have more accurate investigations.

The technique used in this study for flaring with GG was crown-down. Researchers have proven that crown-down sequence is more secure for early preparation purposes because larger instruments tend to penetrate less deeply into the canal, avoiding a great and unnecessary wear of the dentin walls [31]. In this condition, the insertion axis of small drills is altered and this fact allows the smaller drills to shape 2 mm increments of the canal. In other words, this method provides deeper access with smaller drills. Therefore, if the instrument is used correctly, it would be a beneficial, safe and inexpensive tool [15].

In this study, despite random distribution of samples based on Schneider’s method, a considerable difference was observed between groups after SLA preparation. It seems that Schneider’s method is not adequate enough for sample selection purposes as it provides only some information about apical geometry of canal curvature and does not describe the geometry of coronal part of the root canal curvature [7]. On the other hand, spatial configuration, angulation and location of access cavity is different between samples depending on the degree of canal curvature (*i.e*. the angle at which canal separates from pulp chamber), position of root apex against cusp tip, canal length, calcification rate and tooth size, shape and position in the dental arch [7]. As a result, the divergence of access cavity walls produced by elimination of the overhangs of chamber roof is different between samples. Since canal curvature as a confounding variable affects the reliability of results, considering it in future studies, is recommended.

As it was mentioned before, WL in curved canals varies after obtaining SLA. For example, Sadeghi and Doago reported an average change of 0.56 mm. According to them, instead of SS and GG drills, NiTi rotary orifice shapers are suggested for coronal flaring as they result in minimum changes of the WL [7]. Their result is somehow inconsistent with the results of the present study and confirms the results of previous studies. It seems that the less change in WL means the less change in the original shape of canal and root curvature. This leads to satisfactory results in the canals without curves or with slight coronal curvatures while it could result in procedural accidents in cases of canals with greater coronal curvature.

In methodology of the current study the important issue was the emphasis on obtaining a better SLA, *i.e.* selective removal of the thicker and safer segment of root, with special instruments prior to determining the WL and estimation of the actual diameter of apical zone. But almost all of the mentioned studies investigated the variations of the mentioned variables due to the special instrument before and after SLA. As a result, in cases where WL is determined and canal diameter is estimated in post-SLA stage, the more ideal instrument is the one that could give the best SLA and demands the less change of WL and canal diameter estimation.

## Conclusion

Type of instrument plays an important role in gaining straight-line access (SLA). The maximum change of coronal curvature was seen in the Gates Glidden (GG) group. It seems that the risk of unexpected events especially in curved canals could be reduced by using GG drills for obtaining SLA before determining the working length as well as estimating the diameter of the apical zone.
